# Comparison of the Efficacy and Safety of Intravenous Ceftazidime-Avibactam and Intrathecal/Intraventricular Polymyxin B Sulfate in the Treatment of CNS Infections Caused by KPC-*Kp* in Neurosurgical Patients: A Single-Center Prospective Observational Study

**DOI:** 10.3390/antibiotics15050492

**Published:** 2026-05-13

**Authors:** Mei-Hua Wang, Nan-Yang Li, Wei Chen, Ya-Xin Fan, Wan-Zhen Li, Yin-Ru Chen, Jin Hu, Gang Wu, Jing Zhang, Lei Yang

**Affiliations:** 1 Department of Neurosurgery & Neurocritical Care, Huashan Hospital, Fudan University, Shanghai 200040, China; wangmeihua0608@163.com (M.-H.W.); jinhudn2025@163.com (J.H.); woogangng@sina.com (G.W.); 2 Department of Neurosurgery Neurosurgical Institute of Fudan University, Shanghai Clinical Medical Center of Neurosurgery, Shanghai Key Laboratory of Brain Function and Restoration and Neural Regeneration, National Center for Neurological Disorders, Huashan Hospital, Fudan University, Shanghai 200040, China; 3 Clinical Pharmacology Research Center, Huashan Hospital, Fudan University, Shanghai 200040, China; linanyang@fudan.edu.cn; 4 Department of Nursing, Huashan Hospital, Fudan University, 12 Wulumuqi Zhong Road, Shanghai 200040, China; 18721380466@163.com; 5 Institute of Antibiotics, Huashan Hospital, Fudan University, Shanghai 200040, China; fanyaxin@fudan.edu.cn (Y.-X.F.); wanzhen_li@fudan.edu.cn (W.-Z.L.); yinruchen23@m.fudan.edu.cn (Y.-R.C.); 6 Key Laboratory of Clinical Pharmacology of Antibiotics, National Health Commission of the People’s Republic of China, Shanghai 200040, China

**Keywords:** ceftazidime-avibactam, polymyxin B sulfate, *Klebsiella pneumoniae* carbapenemase-producing *K. pneumoniae*, CNS infection, neurosurgery, cerebrospinal fluid, pharmacokinetics, neurotoxicity

## Abstract

**Background:*** Klebsiella pneumoniae* carbapenemase-producing *K. pneumoniae* (KPC-*Kp*) central nervous system (CNS) infections represent a major therapeutic challenge in neurosurgical patients. Intraventricular or intrathecal polymyxin B sulfate (PMB) is commonly used as salvage therapy but is limited by substantial neurotoxicity. Ceftazidime–avibactam (CZA) exhibits potent in vitro activity against KPC-*Kp*; however, prospective clinical and pharmacokinetic evidence supporting its use in CNS infections remains limited. **Methods:** In this prospective, single-centre observational study, adult neurosurgical patients with culture-confirmed KPC-*Kp* CNS infections admitted to the neurointensive care unit of Huashan Hospital were enrolled. Patients received either intravenous CZA (CZA group, *n* = 15) or intrathecal/intraventricular PMB-based therapy (PMB group, *n* = 10). Primary outcomes included clinical cure, microbiological eradication, 28-day mortality, and safety. Therapeutic drug monitoring was performed to determine steady-state plasma and cerebrospinal fluid (CSF) concentrations of ceftazidime, avibactam, and polymyxin B, enabling assessment of CSF penetration and exposure–toxicity relationships. **Results:** Overall clinical cure and microbiological eradication rates were 68.0% and 84.0%, respectively, with a 28-day mortality of 20.0%. Compared with PMB, CZA was associated with a significantly higher clinical cure rate (86.7% vs. 40.0%, *p* = 0.024) and a numerically higher eradication rate (93.3% vs. 70.0%). No neurological adverse events occurred in the CZA group, whereas neurological toxicity was observed in 60.0% of PMB-treated patients (*p* < 0.001). Functional outcomes favoured the CZA group, with lower modified Rankin Scale scores at discharge and at 6 months. Pharmacokinetic analyses demonstrated that steady-state CSF concentrations of ceftazidime and avibactam exceeded commonly accepted pharmacodynamic targets, while markedly elevated PMB CSF concentrations were observed in patients with neurological toxicity. **Conclusions:** While intravenous CZA showed potentially favourable efficacy and safety compared with local PMB in this cohort, these preliminary findings should be interpreted as hypothesis-generating given the small sample size and non-randomised design. These results provide a rationale for further validation in larger multicentre, randomised controlled trials.

## 1. Introduction

*Klebsiella pneumoniae* carbapenemase-producing *K. pneumoniae* (KPC-*Kp*) is recognised as one of the most significant pathogens in neurosurgical patients, particularly those with post-operative central nervous system (CNS) infections. Such infections are associated with high morbidity and mortality, limited treatment options, and a high risk of poor neurological outcomes. Achieving adequate antimicrobial concentrations in the cerebrospinal fluid (CSF) is further complicated by the restrictive nature of the blood–brain barrier (BBB) [1,2]. Despite progress in antimicrobial development, there remains a limited availability of effective and safe therapies with reliable CSF penetration for KPC-*Kp* CNS infections.

Recent studies suggest that ceftazidime-avibactam (CZA) may represent a potential treatment option for KPC-*Kp* CNS infections, particularly in critically ill patients [3,4]. However, the optimal dosing of polymyxin B sulfate (PMB) remains controversial due to its narrow therapeutic index and potential for neurotoxicity [5,6]. Although CZA has been proposed as a promising agent, robust prospective evidence in CNS infections is still lacking, and PMB remains employed as salvage therapy in clinical practice despite its known toxicity profile.

Polymyxins, especially PMB, have been employed intrathecally (ITH) or intraventricularly (IVT) as salvage therapy for KPC-*Kp* CNS infections. Several clinical series reported favourable CSF sterilisation rates when PMB was administered locally with systemic antibiotics [7,8]. However, these regimens are frequently complicated by neurotoxicity, including seizures, encephalopathy, and intracranial haemorrhage, potentially related to high CSF exposure [9,10,11].

CZA, a combination of a third-generation cephalosporin and a non-β-lactam β-lactamase inhibitor, has demonstrated potent in vitro activity against KPC-*Kp*, including KPC-producing strains [12]. While approved for systemic infections such as complicated intra-abdominal and urinary tract infections, its role in CNS infections is less established [13]. Limited case series and PK studies suggest that CZA achieves measurable penetration across the BBB, with CSF/plasma ratios of 30–50% and concentrations exceeding minimum inhibitory concentrations (MICs) for KPC-*Kp* [14,15]. Clinical outcomes from non-CNS KPC-*Kp* infections further indicate that CZA may provide superior efficacy and safety compared with polymyxin-based regimens [16,17]. Nevertheless, evidence for CZA in CNS infections primarily derives from case reports and retrospective studies, with limited prospective data available.

This prospective observational study evaluated the efficacy, microbiological eradication, safety, and cerebrospinal fluid pharmacokinetics of intravenous CZA in neurosurgical patients with KPC-*Kp* CNS infections. A key secondary objective was a head-to-head comparison with intrathecal/intraventricular PMB, including an assessment of CSF penetration and exposure-outcome and exposure-toxicity relationships. We hypothesised that intravenous CZA may be associated with comparable or improved clinical and microbiological outcomes, together with a more favourable safety profile, compared with PMB local therapy in this setting.

## 2. Results

### 2.1. Baseline Demographic, Clinical, and Microbiological Characteristics

**Figure 1:**
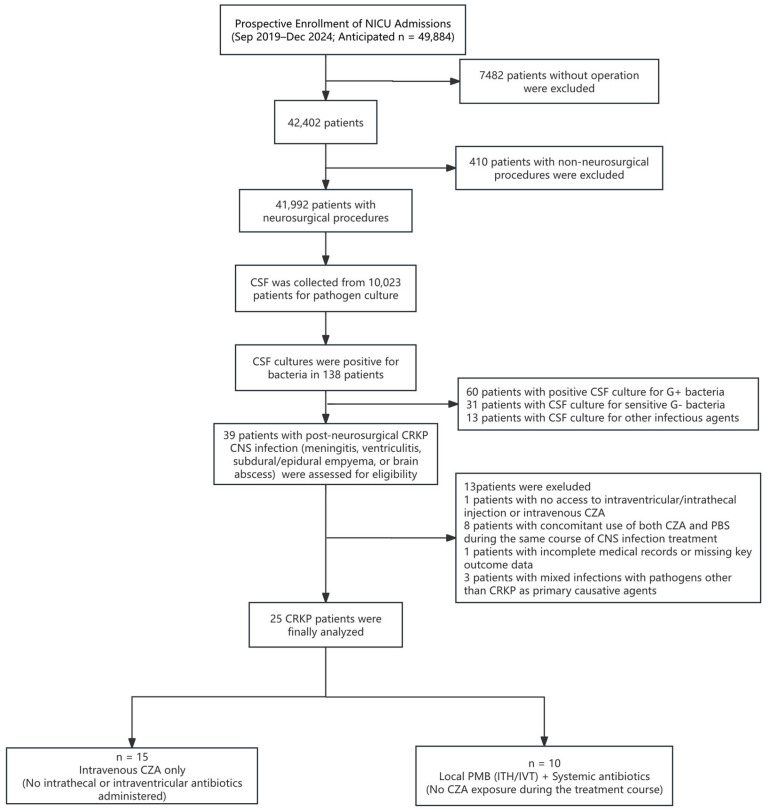
Study flow diagram of patient enrolment.

A total of 25 patients with culture-confirmed KPC-*Kp* central nervous system (CNS) infections were enrolled (Figure 1). The cohort was divided into two treatment groups: 15 patients received intravenous CZA (CZA group), and 10 patients received intrathecal or intraventricular polymyxin B sulfate (PMB group). Baseline characteristics and comparative statistics between groups are presented in Table 1. The study population had a mean age of 44.9 ± 14.2 years and was predominantly male (24/25, 96.0%). The most frequent primary neurosurgical conditions prior to infection were postoperative traumatic brain injury (TBI) in 10 patients (40.0%), postoperative intracranial haemorrhage (ICH) in 6 patients (24.0%), and brain tumour/pituitary adenoma resections in 4 patients each (16.0%). Other primary diagnoses included arteriovenous malformation (AVM, 2/25, 8.0%), cerebral infarction (2/25, 8.0%), and recurrent suprasellar cyst (1/25, 4.0%). Comorbidities included hypertension in 7 patients (28.0%) and diabetes mellitus in 3 patients (12.0%).

At the initial presentation of infections, patients exhibited severe neurological impairment, with median Glasgow Coma Scale (GCS) components of E: 2 (IQR 1–3), M: 3 (IQR 1–5), V: 4 (IQR 1–5), and a median mRS of 3 (IQR 2–5). CSF parameters confirmed severe infection, with markedly elevated WBC (11,736 ± 25,009/μL), protein (4934.6 ± 5830.5 mg/L), and lactate (8.97 ± 4.34 mmol/L). The mean estimated glomerular filtration rate (eGFR) was 132.8 ± 34.6 mL/min/1.73 m^2^. Baseline APACHE II and SOFA scores were 14.68 ± 4.82 and 4.28 ± 1.95, respectively.

### 2.2. Surgical Procedures Before and After CNS Infections

All 25 patients had undergone neurosurgical interventions prior to the onset of KPC-*Kp* CNS infections (Table 1). Most patients had multiple neurosurgical procedures before infections: combined procedures in 17/25 (68.0%) and single procedures in 4/25 (16.0%). Specific operations before infection included neurosurgical craniotomy/intracranial space-occupying resection (6/25, 24.0%), evacuation of intracranial haematoma (7/25, 28.0%), decompressive craniectomy (4/25, 16.0%), and interventional surgery for aneurysm/vascular malformation (3/25, 12.0%). Minimally invasive puncture and drainage procedures were common (15/25, 60.0%). Pre-existing intracranial devices and CSF leakage before infection were also frequent: an Ommaya reservoir in 11/25 (44.0%), intracranial pressure (ICP) monitoring in 9/25 (36.0%), a ventriculoperitoneal shunt (VP) in 4/25 (16.0%), and an external ventricular drain (EVD) in 4/25 (16.0%). Implantable drainage devices were present in 10/25 (40.0%). CSF leak was recorded in 4/25 (16.0%). Detailed surgical and device-related data are provided in Supplementary Table S1.

### 2.3. Systemic Therapy and Microbiological Characteristics

In this cohort of 25 patients, intravenous antimicrobial therapy was dominated by CZA alone (11/25, 44.0%), followed by combinations such as Tigecycline and Amikacin (4/25, 16.0%), while Polymyxin B plus Fosfomycin was administered in 2 patients (8.0%). The remaining patients received other regimens (Table 1). Notably, ten patients received PMB via intrathecal or intraventricular routes. All isolates were confirmed as KPC-*Kp* producing KPC carbapenemase. CZA MICs ranged from 0.25 to 4 mg/L, with most isolates ≤ 2 mg/L.

### 2.4. Clinical and Microbiological Outcomes

**Figure 2:**
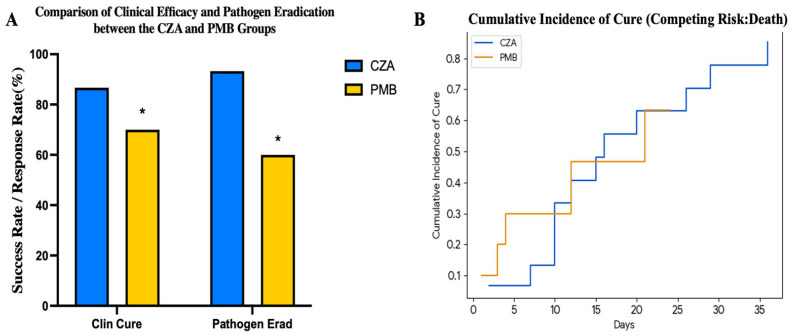
Comparison of clinical efficacy and microbiological outcomes between treatment groups. (**A**) Bar chart depicting the rates of clinical cure and pathogen eradication in patients treated with intravenous ceftazidime-avibactam (CZA group, *n* = 15) versus those treated with intrathecal/intraventricular polymyxin B sulfate (PMB group, *n* = 10). Clinical cure was defined as resolution or significant improvement of infection-related signs/symptoms; pathogen eradication was defined as negative follow-up cerebrospinal fluid culture. Statistical comparisons were performed using the χ^2^ or Fisher’s exact test, as appropriate (* *p* < 0.05). Abbreviations: CZA, ceftazidime-avibactam; PMB, polymyxin B sulfate. (**B**) Cumulative incidence of clinical cure for the CZA and PMB groups, estimated using a Fine-Gray competing risk model with death as the competing event. The cumulative incidence of cure showed a trend towards a higher rate in the CZA group compared to the PMB group (Gray’s test, *p* = 0.051). The blue and orange lines represent the CZA and PMB groups, respectively. Abbreviations: CZA, ceftazidime-avibactam; PMB, polymyxin B sulfate.

Overall, 17/25 patients (68.0%) achieved clinical cure and 21/25 (84.0%) had microbiological eradication (Figure 2A); 28-day mortality was 5/25 (20.0%). Mean time to CSF culture negative results was 8.24 ± 4.53 days, and mean time to clinical cure was 12.76 ± 9.71 days (Table 1).

### 2.5. Comparative Treatment Outcomes and Clinical Efficacy Between the CZA and PMB Groups

At baseline, the PMB group presented with greater neurological impairment as reflected by a significantly higher median admission mRS score (5 [IQR 4–5] vs. 3 [IQR 2–3], *p* < 0.001) and higher CSF lactate levels (11.8 ± 3.7 vs. 7.2 ± 3.7 mmol/L, *p* = 0.010) (Table 1). CSF leakage was more frequent in the CZA group (46.7% vs. 0.0%, *p* = 0.012). Both groups showed elevated renal function. The mean eGFR was elevated in both groups (CZA: 138.5 ± 28.9; PMB: 123.5 ± 41.2 mL/min/1.73 m^2^), with several patients in the CZA group meeting criteria for augmented renal clearance (eGFR > 130 mL/min/1.73 m^2^).

Treatment outcomes appeared more favourable in the CZA group. Clinical cure rates were significantly higher in the CZA group (13/15, 86.7%) than in the PMB group (4/10, 40.0%, *p* = 0.024) (Figure 2A). The cumulative incidence of clinical cure, considering the competing risk of death, also showed a more favourable trend in the CZA group (Figure 2B) (Gray’s test, *p* = 0.051). Microbiological eradication was also significantly higher with CZA (15/15, 100% vs. 6/10, 60.0%, *p* = 0.017).

The mean time to clinical cure was significantly longer in the CZA group (15.67 ± 9.91 days) than in the PMB group (4.75 ± 4.77 days, *p* = 0.004). It should be noted that in the time-to-cure analysis, data from 3 patients in the PMB group who died before achieving clinical cure were censored. The PMB group had a higher early mortality rate (30.0% vs. 13.3% by day 28), resulting in a greater proportion of censored observations in the time-to-cure analysis. The time to CSF culture negative results was comparable (8.1 ± 4.8 vs. 8.5 ± 4.3 days, *p* = 0.832).

Univariate logistic regression analysis (Supplementary Table S2) identified treatment group (OR = 9.75, 95% CI 1.54–61.87, *p* = 0.016) and admission mRS (OR = 0.14, 95% CI 0.02–0.81, *p* = 0.028) as significant predictors of clinical cure.

Subgroup analysis based on the activity of systemic antimicrobial agents (Table 2) further revealed that even among patients receiving active systemic therapy, the clinical cure rate remained significantly lower in the PMB group (44.4% vs. 86.7% in the CZA group, *p* = 0.047), and the pathogen eradication rate was also lower (55.6% vs. 100.0%, *p* = 0.009).

### 2.6. Comparative Safety and Functional Prognosis Between the CZA and PMB Groups

A distinct difference in safety profiles was observed between the two groups. Neurological adverse events occurred exclusively in the PMB group (6/10, 60.0%) and did not occur in the CZA group (*p* < 0.001). Specific complications in the PMB group included paroxysmal sympathetic hyperactivity (6/10, 60.0%), new intracranial haemorrhage (6/10, 60.0%), symptomatic epilepsy (1/10, 10.0%), and new cerebral infarction (1/10, 10.0%) (Table 1). In contrast, no significant nephrotoxicity or hepatotoxicity was observed in either group.

Functional outcomes, assessed by the modified Rankin Scale (mRS), showed significantly better results in the CZA group (Table 1). At discharge, the median mRS was significantly lower in the CZA group (3 [IQR 2–3]) compared with the PMB group (4 [IQR 3–5], *p* = 0.004). This difference in neurological recovery persisted at the 6-month follow-up (3 [IQR 2–4] vs. 4 [IQR 3–5], *p* = 0.026).

### 2.7. Pharmacokinetic Analysis Parameters and BBB Penetration Assessment

**Figure 3:**
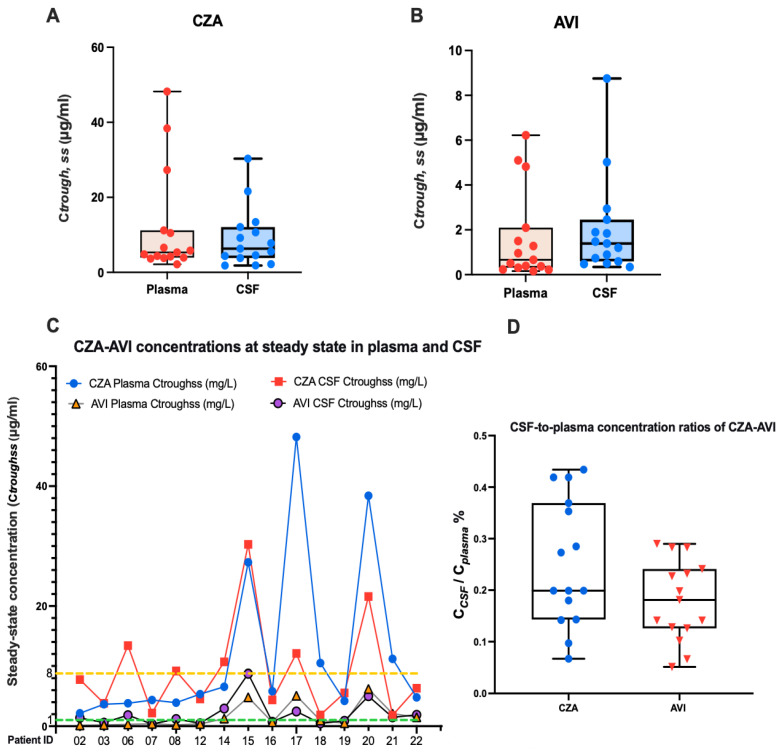
Cerebrospinal fluid exposure and blood–brain barrier penetration of ceftazidime and avibactam. (**A**,**B**) Steady-state trough concentrations (C_trough,ss_) of ceftazidime (CAZ, panel (**A**)) and avibactam (AVI, panel (**B**)) in plasma and cerebrospinal fluid (CSF). Horizontal bars denote the median values for each compartment. (**C**) Individual patient steady-state trough concentrations (C_trough,ss_) of AVI in plasma and CSF, showing their distribution relative to its pharmacodynamic target. The dashed horizontal lines indicate the predefined pharmacodynamic target values (CAZ MIC = 8 mg/L, orange line; AVI target concentration = 1 mg/L, green line), selected based on published PK/PD benchmarks. (**D**) CSF-to-plasma penetration ratios of CAZ and AVI, calculated using steady-state concentrations (average of trough and peak). Boxes represent the median and interquartile ranges, with whiskers indicating the full range of observed values, illustrating inter-patient variability in CNS penetration. Abbreviations: CAZ, ceftazidime; AVI, avibactam; CSF, cerebrospinal fluid; CNS, central nervous system; C_trough,ss_, trough concentration at steady state; MIC, minimum inhibitory concentration; PK/PD, pharmacokinetic/pharmacodynamic;.

For CAZ (Figure 3A) and AVI (Figure 3B), measurable concentrations were achieved in both plasma and cerebrospinal fluid (CSF) at steady state. The mean steady-state trough concentrations (C_trough,ss_) for CAZ were 11.58 mg/L in plasma and 9.12 mg/L in CSF, and for AVI were 1.68 mg/L and 2.07 mg/L, respectively (individual pharmacokinetic parameters are detailed in Table 3). Critically, the mean C_trough,ss_ in CSF exceeded the respective pharmacodynamic targets (8 mg/L for CAZ and 1 mg/L for AVI). At the individual patient level (Figure 3C), the CSF C_trough,ss_ of CAZ was ≥8 mg/L in 6/15 (40.0%) of patients, while that of AVI was ≥1 mg/L in 9/15 (60.0%) of patients. The blood–brain barrier penetration, quantified as the CSF-to-plasma concentration ratio based on steady-state average concentrations (Figure 3D), demonstrated a median value of approximately 25.2% for CAZ and 18.4% for AVI. The correlation between PK/PD target attainment and clinical outcomes in the CZA group is detailed in Table 4.

### 2.8. Therapeutic Drug Monitoring of PMB Concentration in Cerebrospinal Fluid and Neurotoxicity

**Figure 4:**
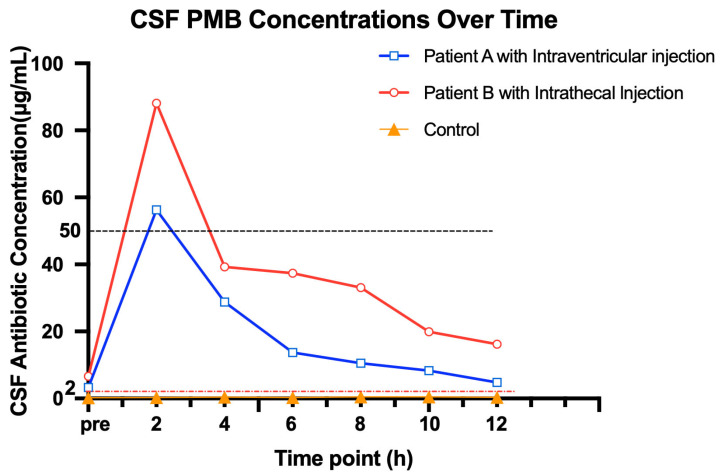
Cerebrospinal fluid concentrations of polymyxin B sulfate and correlation with neurotoxicity. (Peak polymyxin B concentrations in the cerebrospinal fluid (CSF) following intraventricular or intrathecal administration of polymyxin B sulfate (PMB) in three representative patients. The horizontal dashed line represents the typical susceptibility breakpoint (2 mg/L), highlighting the markedly supratherapeutic drug exposure achieved. Yellow triangles represent the control patient who did not receive local PMB administration (CSF concentration = 0 mg/L). Abbreviations: PMB, Polymyxin B Sulfate; CSF, Cerebrospinal Fluid.

The notably high incidence of neurological adverse events in the PMB group can be mechanistically explained by excessive drug exposure in the cerebrospinal fluid (CSF). Pharmacokinetic (PK) monitoring in PMB-treated patients (Figure 4 and Supplementary Table S3) revealed that peak polymyxin B concentrations in the CSF were extremely high, with a median peak concentration of 64.2 mg/L (range: 48.5–88.0 mg/L). These concentrations far exceeded the MIC for KPC-*Kp* (typically ≤2 mg/L), indicating consistently toxic drug levels. Importantly, these elevated concentrations were temporally correlated with the onset of neurological toxicity. These results suggest that concentration-dependent neurotoxicity may contribute substantially to PMB’s adverse safety profile.

## 3. Discussion

To our knowledge, this prospective study is among the limited available reports that directly compare intravenous CZA with intrathecal or intraventricular PMB in critically ill neurosurgical patients with KPC-*Kp* CNS infections. In our study, intravenous CZA was associated with significantly higher clinical cure and microbiological eradication rates compared to PMB. Furthermore, subgroup analysis confirmed that even among patients receiving active systemic backbone therapy (e.g., tigecycline or amikacin), the clinical cure rate in the PMB group remained substantially lower than that in the CZA group, suggesting that sub-optimal systemic activity in the PMB group does not fully explain the observed efficacy gap.

First, steady-state concentrations of CZA were detectable in the cerebrospinal fluid (CSF) of a subset of neurosurgical patients following intravenous administration. The mean steady-state trough concentrations (C_trough,ss_) for ceftazidime were 11.58 mg/L in plasma and 9.12 mg/L in CSF, and for avibactam were 1.68 mg/L and 2.07 mg/L, respectively. Although CSF-to-plasma ratios remained below 0.5, the CSF C_trough,ss_ exceeded the respective pharmacodynamic targets in a substantial proportion of patients: 40.0% achieved CAZ C_trough,ss_ ≥ 8 mg/L, and 60.0% achieved AVI C_trough,ss_ ≥ 1 mg/L KPC-*Kp*. These findings suggest that standard dosing of CZA can provide pharmacodynamically relevant exposure within the CSF compartment, supporting its clinical efficacy.

The median CSF-to-plasma concentration ratios were approximately 21% for CAZ and 26% for AVI, indicating moderate blood–brain barrier penetration. Despite these relatively low ratios, the achieved CSF concentrations were sufficient to exceed pharmacodynamic thresholds in most patients, reinforcing the potential for systemic CZA therapy to attain effective CNS exposure.

Avibactam exhibited lower absolute CSF concentrations than ceftazidime; however, as a β-lactamase inhibitor, it does not require intrinsic bactericidal activity. Rather, its function is to preserve ceftazidime activity by inhibiting KPC-mediated hydrolysis. The CSF avibactam concentrations observed in this study, though modest, are likely sufficient to inhibit β-lactamases within the CNS milieu, thereby maintaining ceftazidime efficacy. This mechanistic consideration may partly explain the favourable microbiological outcomes despite lower avibactam CSF exposure.

Notably, the time to clinical cure was longer in the CZA group, a finding that likely reflects the more severe baseline neurological impairment and higher early mortality in the PMB group, rather than differences in intrinsic antimicrobial activity [18]. Although CZA was associated with a higher overall clinical cure rate, patients in the PMB group had markedly worse baseline neurological function and a higher rate of early mortality, resulting in a larger proportion of censored observations. Early deaths can artificially shorten the estimated mean time to clinical cure in survival-type analyses, a well-recognised limitation in non-randomised cohorts [19]. This is further supported by our cumulative incidence function, which shows that CZA’s efficacy is sustained over time despite a more complex clinical course.

Second, patients treated with intraventricular/intrathecal polymyxin B experienced a high incidence of neurological adverse events, including intracranial haemorrhage, which coincided with supratherapeutic CSF polymyxin concentrations. These findings suggest that PMB should be used with caution, possibly reserved for salvage therapy and administered under strict PK guidance. Importantly, the incidence of intracranial haemorrhage in our cohort was higher than previously reported, which may be associated with multiple factors, including (1) markedly elevated CSF PMB levels, (2) the severe neurological status at baseline, and (3) the presence of invasive intracranial devices, all known contributors to haemorrhagic complications [20,21]. Taken together, these findings indicate that intravenous CZA may represent a safer systemic option for KPC-*Kp* CNS infections, while the role of local polymyxin administration warrants careful reconsideration.

The favourable outcomes associated with intravenous CZA are consistent with previous studies demonstrating superior safety and clinical performance compared with polymyxin-based regimens in bloodstream and pulmonary infections [22,23]. Although reports of CZA for CNS infections have largely been limited to case series or retrospective cohorts [4,24], our prospective data add to the emerging evidence supporting its CNS use.

Our study also highlights the considerable risks associated with local PMB administration. Neurological adverse events occurred in 60% of PMB-treated patients, including intracranial haemorrhage in 60%. These rates surpass those reported in pooled analyses [25], and our PK findings indicate markedly elevated CSF polymyxin B concentrations (up to 88 mg/L), compatible with concentration-dependent neurotoxicity described in experimental models [26]. These observations support the notion that the therapeutic window of intrathecal or intraventricular polymyxins is extremely narrow and characterised by unpredictable CSF accumulation and substantial safety concerns [27]. This toxicity profile appears to be related to the pharmacokinetic behaviour of PMB in the CNS compartment.

Patients in the PMB group exhibited significantly greater baseline neurological impairment and illness severity, introducing “confounding by indication,” a limitation inherent to non-randomised designs. Nonetheless, even when considering these differences, the disparity in safety and functional outcomes remained clinically relevant. The relatively rapid CSF sterilisation observed in the PMB group likely reflects higher early mortality and truncated treatment courses, rather than more rapid microbiological clearance. This should be interpreted in the context of higher baseline SOFA scores, elevated CSF protein, and the need for urgent local interventions. These factors likely contributed to shorter apparent sterilisation times, underscoring the need for careful adjustment for baseline severity in comparative analyses.

Both groups demonstrated increased eGFR, with several patients in the CZA group exhibiting values consistent with augmented renal clearance (ARC). ARC is increasingly recognised in critically ill patients, including neurosurgical populations, and may reduce plasma and CSF concentrations of hydrophilic antimicrobials such as CZA [28]. In our cohort, the elevated mean eGFR was consistent with augmented renal clearance (ARC), a condition known to accelerate the systemic elimination of hydrophilic antimicrobials like CZA. This phenomenon potentially diminishes the plasma-to-CSF concentration gradient required for effective blood–brain barrier (BBB) penetration. Furthermore, while our study primarily focused on diffuse meningitis, target attainment may vary in the presence of localised infection foci, such as brain abscesses or infected surgical wounds, which were observed in a subset of our patients. These factors, combined with ARC, represent critical confounders necessitating individualised dosing strategies guided by TDM. This highlights the importance of individualised dosing and therapeutic drug monitoring to ensure adequate CNS exposure, particularly in patients with ARC. Recent PK modelling studies also support the utility of TDM-guided optimisation of CZA dosing in settings of variable renal clearance [29].

In terms of therapeutic strategy, intravenous CZA may be considered as a systemic option for KPC-*Kp* CNS infections when isolates are susceptible, with TDM incorporated to optimise systemic and CNS exposure. In contrast, intraventricular/intrathecal PMB is best reserved for salvage therapy in carefully selected cases, administered with caution and ideally guided by PK monitoring. Combination regimens, such as CZA with low-dose polymyxin or fosfomycin, may be useful in refractory cases, though supporting evidence remains limited and such approaches should be implemented within controlled clinical frameworks [30]. Ultimately, treatment strategies that prioritise systemically administered agents with reliable BBB penetration may contribute to improved survival and neurological outcomes in this vulnerable population.

This study has limitations. It was conducted at a single centre with a small sample size and a non-randomised design, which may limit generalisability and leave residual confounding. Specifically, the PMB group exhibited greater baseline neurological impairment (higher mRS scores). Due to the limited sample size, we performed only univariate analyses. We also employed a competing risk model and subgroup analysis to explore potential biases. Nevertheless, the wide confidence intervals reflect limited statistical power, and residual confounding cannot be entirely excluded. Furthermore, PK data were available only for a subset of patients in the CZA group, and long-term outcomes beyond six months, such as recurrence and resistance development, were not comprehensively evaluated. Larger, multicentre prospective studies, ideally with randomised or propensity-matched designs, are needed to validate these findings, refine dosing strategies, and clarify the role of adjunctive therapies.

In summary, this prospective study provides preliminary comparative evidence evaluating intravenous CZA versus intrathecal or intraventricular PMB in post-neurosurgical KPC-*Kp* CNS infections. Intravenous CZA achieved sustained CSF exposure above MICs, high clinical and microbiological cure rates, and a favourable safety profile. By contrast, PMB was associated with supratherapeutic CSF accumulation and significant neurotoxicity, including intracranial haemorrhage.

## 4. Materials and Methods

### 4.1. Study Design and Setting

This prospective, single-centre observational study was conducted in the neurosurgical intensive care unit (NICU) of Huashan Hospital, Fudan University. Patients were enrolled from September 2019 to December 2024 (Figure 1). Patients were treated with one of two therapeutic regimens, as determined by the attending physicians in consultation with infectious disease specialists, based on antimicrobial susceptibility, drug availability, and the overall clinical context, and were not adjusted based on real-time pharmacokinetic data. The study was approved by the Ethics Committee of Huashan Hospital (No. 2019-475), and written informed consent was obtained from all participants or their legal surrogates prior to enrolment. This report followed the STROBE guidelines for observational studies.

This diagram illustrates the process of screening, enrolment, and allocation of patients with culture-confirmed KPC-*Kp* central nervous system (CNS) infections into the two treatment groups: intravenous ceftazidime-avibactam (CZA) and intrathecal or intraventricular polymyxin B sulfate (PMB). Systemic antibiotics in the PMB group were individualised based on susceptibility (excluding CZA), as detailed in Supplementary Table S1.

### 4.2. Inclusion and Exclusion Criteria

Adult patients aged 18 years or older who had undergone neurosurgical procedures and subsequently developed KPC-*Kp* CNSinfections were screened for eligibility. The diagnosis of post-neurosurgical CNS infection was established according to the 2017 Infectious Diseases Society of America (IDSA) clinical practice guidelines for healthcare-associated ventriculitis and meningitis [1]. For patients presenting with brain abscess or empyema, diagnosis was confirmed by characteristic neuroimaging findings (CT or MRI showing ring-enhancing lesions or focal fluid collections) in conjunction with clinical manifestations [1]. Only patients with positive cerebrospinal fluid (CSF) cultures for *Klebsiella pneumoniae* and antimicrobial susceptibility testing confirming carbapenem resistance, defined as resistance to meropenem or imipenem according to Clinical and Laboratory Standards Institute (CLSI) criteria, were included. In addition, eligible patients presented with compatible clinical manifestations such as fever, altered consciousness, or meningeal signs, accompanied by abnormal CSF findings including pleocytosis, elevated protein levels, or decreased glucose concentrations.

Patients were excluded if they had non-KPC-*Kp* infections or mixed infections where KPC-*Kp* was not the predominant pathogen; known severe hypersensitivity to CZA or polymyxin B sulfate and their excipients; severe and uncorrectable hepatic or renal dysfunction that might significantly affect drug metabolism or increase toxicity risk; or were unable to undergo intraventricular or intrathecal administration due to anatomical abnormalities, lack of external ventricular drainage devices, or high bleeding risk. Additional exclusion criteria included an expected survival time of less than 72 h due to other life-threatening comorbidities, pregnancy or lactation, and incomplete or missing key clinical information such as CSF culture and susceptibility results, treatment details, or outcome data. Patients who died within 48 h of infection onset were also excluded.

### 4.3. Treatment Groups

In the CZA group, patients received exclusively intravenous (IV) CZA (2.5 g; 2 g ceftazidime plus 0.5 g avibactam) infused over at least 2 h every 8 h. For patients exhibiting augmented renal clearance (ARC), defined as an eGFR > 130 mL/min/1.73 m^2^ [31], the dosage was maintained at 2.5 g every 8 h, but administered via prolonged infusion (over 3 h) to optimise pharmacodynamic target attainment, consistent with established PK/PD principles for hydrophilic β-lactams in critically ill populations [32]. No intrathecal (ITH) or intraventricular (IVT) antibiotics were administered in this group. The dose, frequency, and duration of treatment were predefined in the study protocol to ensure treatment consistency.

In the PMB group, patients received local administration (ITH or IVT) of polymyxin B sulfate and did not receive CZA at any point during the treatment course for this CNS infection. Administration was performed according to local neurosurgical protocols [33]. The typical starting dose was 5 mg once daily, with adjustments (range, 5–10 mg/day) based on body weight, infection severity, clinical response, CSF sterilisation, and tolerability. PMB was administered in combination with individualised systemic antimicrobial regimens (excluding CZA) selected according to susceptibility profiles (detailed in Supplementary Table S1), which were based on local epidemiological data and evidence of synergistic activity. Administration adhered to standard aseptic procedures, including temporary clamping of CSF drainage before and after injection, slow injection of the drug, and careful post-injection site management. Neurological adverse events were closely monitored throughout treatment. PMB dosing strategies were standardised according to the study protocol.

### 4.4. Surgical Intervention

Regarding post-infection surgical management, prompt source control was undertaken for patients with infection-related factors that did not adequately respond to medical therapy, including poor wound healing, persistent cerebrospinal fluid leakage, pre-existing external ventricular drainage, or implanted intracranial devices, and prompt surgical management was undertaken. This included removal of infected or potentially contaminated devices when clinically indicated. Detailed information regarding the pre-existing surgical procedures and implanted devices for each patient is provided in Supplementary Table S1.

Separately, to establish a route for patients requiring local antimicrobial administration, new external ventricular drainage (EVD) catheters or Ommaya reservoirs were placed by neurosurgeons under sterile conditions to facilitate intraventricular or intrathecal therapy. In patients who were considered unstable or unsuitable for operative intervention, bedside lumbar drainage was performed by attending physicians to ensure adequate CSF diversion and local drug delivery.

### 4.5. Outcomes

The primary outcomes were clinical cure, microbiological eradication, and the incidence of adverse events, including neurological adverse events. Clinical cure was defined as the resolution or significant improvement of clinical signs and symptoms related to central nervous system infection without the need for additional antibacterial therapy. Microbiological eradication was defined as the absence of the initial causative organism in cerebrospinal fluid (CSF) cultures following completion of treatment.

Neurological adverse events were defined as newly developed or clearly worsened neurological manifestations occurring during treatment, including seizures, encephalopathy, altered consciousness, focal neurological deficits, or new intracranial haemorrhage, as assessed by the treating physicians and confirmed by clinical evaluation and/or neuroimaging where appropriate.

Secondary outcomes included 28-day all-cause mortality, functional outcomes assessed using the modified Rankin Scale (mRS) [34] at discharge and at 6 months, time to clinical cure, and time to CSF culture sterilisation.

### 4.6. Data Collection

Prospectively collected clinical data included demographics, comorbidities, neurosurgical diagnosis, baseline severity scores (SOFA and APACHE II), laboratory indices, treatment regimens, surgical interventions, and supportive therapies. Additional parameters recorded were estimated glomerular filtration rate (eGFR) and requirement for mechanical ventilation. Two investigators independently verified all outcome and follow-up data.

### 4.7. Microbiological Analysis

KPC-*Kp* isolates were extracted from the CSF specimens drained from the ventricle or lumbar puncture. Identification was performed using MALDI-TOF MS (Bruker Daltonics, Bremen, Germany) and the VITEK 2 COMPACT system (BioMérieux, Marcy-l’Étoile, France). Antimicrobial susceptibility testing was conducted by the broth dilution method, measuring ceftazidime MICs in the presence of 4 mg/L avibactam, with concentrations ranging from 0.25 to 128 mg/L. Results were interpreted according to CLSI guidelines [35].

### 4.8. Pharmacokinetic Analysis and Penetration Evaluation

CZA Group: To minimise the infection risk during sampling, plasma and CSF samples were collected from all patients at only two time-points (trough and peak concentration points) 72 h after starting intravenous ceftazidime-avibactam therapy when a steady state was reached [36]. Peak concentration samples were obtained immediately at the end of the 2 h CZA infusion, while trough samples were collected within 30 min prior to the subsequent dose. Drug concentrations were measured by a validated liquid chromatograph–tandem mass spectrometry method. Due to the lack of half-life (T1/2) and area under the concentration-time curve (AUC) data, the percentage of the dosing interval during which the free drug concentration remains above the minimum inhibitory concentration (%fT > MIC) could not be calculated. In this case, the steady-state trough concentration can be carefully used as a practical surrogate indicator to roughly assess the risk of achieving the PK/PD target (defined as 100% fT > 8 times MIC for ceftazidime and C_T_ > 1 mg/L for avibactam) [37,38]. The blood–brain barrier penetration of ceftazidime-avibactam can be approximately estimated by the ratio of the CSF steady-state drug concentration to the synchronized blood steady-state drug concentration (defined as the average of trough and peak concentration).

PMB Group: To characterise drug exposure after local administration, CSF samples were collected from three patients at multiple time points before and after intraventricular/intrathecal polymyxin B injection (e.g., pre-dose, and 1, 2, 4, 6, 8, 10, 12 h post-dose) to depict the concentration-time profile and analyse peak-trough concentrations. The measured CSF polymyxin B concentrations were descriptively evaluated in relation to the occurrence of neurological adverse events to explore potential exposure-related neurotoxicity. This analysis aimed to elucidate the mechanism behind the high incidence of neurological events observed in this cohort.

### 4.9. Statistical Analysis

Continuous variables are presented appropriately as mean ± SD or median (IQR) based on their distribution. The normality of continuous variables was assessed using the Shapiro–Wilk test. Categorical variables are summarised as counts and percentages. Between-group comparisons are performed using Student’s *t* test or the Mann–Whitney U test for continuous variables, depending on whether the data met the assumptions of normality, and χ^2^ or Fisher’s exact test for categorical variables. A Fine-Gray competing risk model was used to compare the cumulative incidence of clinical cure, treating death as a competing event, and the results are presented as cumulative incidence curves. Subgroup analyses were conducted based on the activity of systemic antimicrobial backbones. Because this study was exploratory, no adjustments were made for multiple comparisons. All analyses were performed on the available data without imputation for missing values. A two-tailed *p* value < 0.05 was considered statistically significant. Analyses were performed using SPSS version 26.0 (IBM Corp., Armonk, NY, USA) and R software (version 4.2.0) for the competing risk model.

## 5. Conclusions

This prospective study indicates that intravenous CZA achieves measurable cerebrospinal fluid concentrations and is generally well tolerated, suggesting it may be a feasible treatment option for post-neurosurgical KPC-*Kp* CNS infections when the isolate is susceptible. In contrast, intrathecal or intraventricular polymyxin B was associated with high CSF concentrations and notable neurotoxicity. These observations highlight the potential advantages of systemic agents with blood–brain barrier penetration and suggest that local polymyxin therapy should be reserved for selected cases under careful monitoring. Further multicentre randomised studies are needed to more comprehensively evaluate these findings.

## Figures and Tables

**Table 1:** Clinical Characteristics and Clinical Outcomes of Patients with KPC-*Kp* CNS Infections.

Variables	Total (*N* = 25)	CZA Group (*n* = 15)	PMB Group (*n* = 10)	*p*-Value
Demographics				
Age, years, mean ± SD	44.9 ± 14.2	45.9 ± 13.4	43.4 ± 15.7	0.661
Sex, male, *n* (%)	24 (96.0)	14 (93.3)	10 (100.0)	1
Primary Disease, *n* (%)				
Postoperative ICH	6 (24.0)	5 (33.3)	1 (10.0)	0.35
Postoperative TBI	10 (40.0)	5 (33.3)	5 (50.0)	0.68
Brain tumour/Pituitary adenoma	4 (16.0)	2 (13.3)	2 (20.0)	1
Others (AVM, infarction, etc.)	5 (20.0)	3 (20.0)	2 (20.0)	–
Comorbidities, *n* (%)				
Hypertension	7 (28.0)	5 (33.3)	2 (20.0)	0.667
Diabetes mellitus	3 (12.0)	1 (6.7)	2 (20.0)	0.548
Baseline Severity				
Admission GCS, median (IQR)				
Eye	2 (1, 3)	3 (1, 4)	1.5 (1, 2)	0.076
Motor	3 (1, 5)	4 (2, 5)	2.5 (1, 3)	0.076
Verbal	4 (1, 5)	4 (1, 5)	4 (1, 5)	0.836
Admission mRS, median (IQR)	3 (2, 5)	3 (2, 3)	5 (4, 5)	<0.001
SOFA score, mean ± SD	4.28 ± 1.95	3.5 ± 1.1	5.6 ± 2.2	0.004
APACHE II, mean ± SD	14.68 ± 4.82	13.9 ± 4.8	15.9 ± 4.9	0.304
CSF Parameters, mean ± SD				
Glucose, mmol/L	2.14 ± 1.37	2.3 ± 1.6	1.9 ± 0.9	0.452
WBC,/μL	11,736 ± 25,009	11,669 ± 26,318	11,833 ± 24,282	0.986
Protein, mg/L	4935 ± 5831	3617 ± 4846	7044 ± 6939	0.124
Lactate, mmol/L	8.97 ± 4.34	7.2 ± 3.7	11.8 ± 3.7	0.01
eGFR, mL/min/1.73 m^2^	132.8 ± 34.6	138.5 ± 28.9	123.5 ± 41.2	0.266
Interventions & Devices				
Implants before infection, *n* (%)	10 (40.0)	5 (33.3)	4 (50.0)	0.673
CSF leakage, *n* (%)	11 (44.0)	9 (60.0)	2 (44.0)	0.096
EVD after infection, *n* (%)	12 (48.0)	6 (40.0)	6 (60.0)	0.432
Ommaya after infection, *n* (%)	16 (64.0)	10 (66.7)	6 (60.0)	1
Antimicrobial Treatments				
Local PMB therapy, *n* (%)	10 (40.0)	0 (0.0)	10 (100.0)	<0.001
Dosage, mg/day	–	N/A	5	–
Duration, days, median (range)	–	N/A	6 (3–15)	–
Clinical Outcomes				
Clinical cure, *n* (%)	17 (68.0)	13 (86.7)	4 (40.0)	0.024
Time to cure, days, mean ± SD	12.76 ± 9.71	15.67 ± 9.91	4.75 ± 4.77	0.004
Time to CSF negative, days, mean ± SD	8.24 ± 4.53	8.1 ± 4.8	8.5 ± 4.3	0.832
Pathogen eradication, *n* (%)	21 (84.0)	15 (100.0)	6 (60.0)	0.017
28-day mortality, *n* (%)	5 (20.0)	2 (13.3)	3 (30.0)	0.355
Safety & Functional Prognosis				
Neurological adverse events, *n* (%)	6 (24.0)	0 (0.0)	6 (60.0)	<0.001
Mechanical ventilation, *n* (%)	14 (56.0)	6 (40.0)	8 (80.0)	0.099
Hospital stay, days, mean ± SD	39.4 ± 24.2	46.1 ± 25.8	29.6 ± 20.4	0.097
Discharge mRS, median (IQR)	3 (3, 5)	3 (2, 3)	4 (3, 5)	0.004
6-month mRS, median (IQR)	3 (3, 4)	3 (2, 4)	4 (3, 5)	0.026

Abbreviations: CZA, ceftazidime-avibactam; PMB, polymyxin B; KPC-*Kp*, KPC-producing *Klebsiella pneumoniae*; CNS, central nervous system; SD, standard deviation; ICH, intracranial haemorrhage; TBI, traumatic brain injury; AVM, arteriovenous malformation; GCS, Glasgow Coma Scale; IQR, interquartile range; mRS, modified Rankin Scale; CSF, cerebrospinal fluid; WBC, white blood cell; eGFR, estimated glomerular filtration rate; EVD, external ventricular drain; APACHE, Acute Physiology and Chronic Health Evaluation; SOFA, Sequential Organ Failure Assessment.

**Table 2:** Subgroup Analysis of Clinical Outcomes Based on the Activity of Systemic Antimicrobial Agents (Stratified within the PMB Group).

Treatment Regime	Total (*N*)	Clinical Cure, *n* (%)	Pathogen Eradication, *n* (%)
CZA Group (Active systemic therapy)	15	13 (86.7%)	15 (100.0%)
PMB Group	10	4 (40.0%)	6 (60.0%)
PMB Group Subgroup:			
With Active Systemic Antibacterial Drugs ^a^	9	4 (44.4%)	5 (55.6%)
With Inactive Systemic Antibacterial Drugs ^b^	1	0 (0.0%)	1 (100.0%)
*p*-value ^c^ (CZA vs. PMB Active Subgroup)		0.047	0.009

Abbreviations: CZA, ceftazidime-avibactam; PMB, polymyxin B; KPC-*Kp*, KPC-producing *Klebsiella pneumoniae*; *n*, number. ^a^ Defined as systemic regimens containing at least one agent with potential in vitro activity against KPC-*Kp* (e.g., tigecycline, amikacin, intravenous fosfomycin, or ceftazidime-avibactam). ^b^ Defined as systemic regimens consisting of agents with known resistance or lack of target activity (e.g., standard-dose carbapenems or cephalosporins). ^c^ *p*-value calculated using Fisher’s exact test comparing the CZA group with the PMB-active subgroup.

**Table 3:** Individual Patient Steady-State Pharmacokinetic Parameters of Ceftazidime and Avibactam.

Parameter	Statistic	CZA Plasma C_trough,ss_ (mg/L)	CZA CSF C_trough,ss_ (mg/L)	AVI Plasma C_trough,ss_ (mg/L)	AVI CSF C_trough,ss_ (mg/L)	CZA Penetration Ratio (%)	AVI Penetration Ratio (%)
Sample Size	*n*	15	15	15	15	15	15
Mean (SD)	Mean (SD)	11.58 (13.86)	9.12 (7.94)	1.68 (1.94)	2.07 (2.29)	25.2 (11.7)	18.4 (8.3)
Median (IQR)	Median (IQR)	5.35 (4.02–27.30)	6.33 (3.95–11.20)	0.667 (0.369–2.10)	1.39 (0.738–2.46)	19.9 (14.3–36.9)	18.1 (12.6–24.1)
Minimum	Min	2.15	1.82	0.159	0.342	6.7	5.1
Maximum	Max	48.2	30.3	6.22	8.75	43.4	29
Range	Range	46.05	28.48	6.06	8.41	36.7	23.9
Target Attainment Rate	*n*/*N* (%)	—	6/15 (40.0%)	—	9/15 (60.0%)	—	—

*n*: Sample size (number of patients); Mean: Arithmetic average of the measured values; SD: Standard Deviation, a measure of data dispersion around the mean; Median: The middle value in an ordered dataset; IQR: Interquartile Range, the range between the 25th and 75th percentiles representing the middle 50% of the data; Min: The minimum observed value; Max: The maximum observed value; Range: The absolute difference between the maximum and minimum values; Target Attainment Rate: The proportion of patients whose steady-state trough concentration in cerebrospinal fluid (CSF C_trough,ss_) reached or exceeded the predefined pharmacodynamic target; Penetration Ratio (%): A measure of blood–brain barrier penetration, calculated as (Steady-state CSF Concentration/Concurrent Steady-state Plasma Concentration) × 100%; C_trough,ss_: Steady-state trough concentration, measured immediately before the next dose at pharmacokinetic steady state.

**Table 4:** Correlation between CSF PK/PD Target Attainment and Clinical Outcomes in the CZA Group (*N* = 15).

PK/PD Target Attainment	Clinical Cure (*n* = 13)	Non-Cure/Death (*n* = 2) ^a^	*p*-Value ^b^
CAZ CSF Target (≥8 mg/L)			0.595
Attained (*n* = 6)	5 (83.3%)	1 (16.7%)	
Not Attained (*n* = 9)	8 (88.9%)	1 (11.1%)	
AVI CSF Target (≥1 mg/L)			0.173
Attained (*n* = 9)	7 (77.8%)	2 (22.2%)	
Not Attained (*n* = 6)	6 (100.0%)	0 (0.0%)	

Abbreviations: CSF, cerebrospinal fluid; PK/PD, pharmacokinetic/pharmacodynamic; CAZ, ceftazidime; AVI, avibactam; CZA, ceftazidime-avibactam. ^a^ Non-cure/death patients include CA15 and CA21 (both 28-day mortality). ^b^ Calculated using Fisher’s exact test.

## Data Availability

Lei Yang, the corresponding author, had full access to all the data in the study and takes responsibility for the integrity of the data and the accuracy of the data analysis. Data supporting the findings of this study are available from the corresponding author upon reasonable request.

## Supplementary Materials

The following supporting information can be downloaded at: https://www.mdpi.com/article/10.3390/antibiotics15050492/s1, Table S1: Additional Clinical Characteristics of the Entire Cohort (*N* = 25); Table S2: Univariate Logistic Regression Analysis of Factors Associated with Clinical Cure; Table S3: CSF concentrations of polymyxin B following different administration routes; Table S4: Polymyxin B Sulfate (PBS) Dosing Regimens in Included Patients.
