# Supplementary material for: Comparison of the Efficacy and Safety of Intravenous Ceftazidime-Avibactam and Intrathecal/Intraventricular Polymyxin B Sulfate in the Treatment of CNS Infections Caused by KPC-Kp in Neurosurgical Patients: A Single-Center Prospective Observational Study
Source: Antibiotics (Basel). 2026 May 13;15(5):492. doi: 10.3390/antibiotics15050492 (PMC13203131; doi:10.3390/antibiotics15050492)
Supplement: Supplementary file 1 [file antibiotics-15-00492-s001.zip › new-Supplementary Table S1.pdf]

Supplementary Table S1. Additional Clinical Characteristics of the Entire Cohort (N=25)

Variables	Value
Treatment duration, days, mean ± SD	15.8 ± 7.8
Surgical treatment before intracranial infection, n (%)	
Combined procedures	17 (68.0)
Single procedures	4 (16.0)
Neurosurgical craniotomy / intracranial space occupying resection	6 (24.0)
Evacuation of intracranial hematoma	7 (28.0)
Decompressive craniectomy	4 (16.0)
Intracranial space occupying resection	4 (16.0)
Interventional surgery for aneurysm / vascular malformations	3 (12.0)
Invasive puncture and drainage	15 (60.0)
External ventricular drain (EVD) before infection	4 (16.0)
Intracranial pressure (ICP) monitor	9 (36.0)
Ommaya reservoir before infection	11 (44.0)
Ventriculoperitoneal (VP) shunt	4 (16.0)
Lumbar puncture (LP)	5 (20.0)
Implants before infection, n (%)	
Drainage device	10 (40.0)
Skull repair material or artificial dura	2 (8.0)
Cerebrospinal fluid leakage, n (%)	
Incisional CSF leakage	11 (44.0)
Surgical treatment after intracranial infection, n (%)	
Lumbar cistern drainage	6 (24.0)
Wound repair	4 (16.0)

Systemic antibiotic therapy (intravenous), n (%)	
CZA alone	11 (44.0)
PMB + CZA	1 (4.0)
Amikacin + CZA	2 (8.0)
Tigecycline + Amikacin	4 (16.0)
Polymyxin + Fosfomycin	2 (8.0)
Cefepime + Ciprofloxacin	1 (4.0)
Fosfomycin (IV)	1 (4.0)
Meropenem + Amoxicillin	1 (4.0)
Meropenem + Amoxicillin + Fosfomycin	1 (4.0)
Meropenem + Vancomycin	1 (4.0)
Complications, n (%)	7 (29.0)

Abbreviations: SD, standard deviation; EVD, external ventricular drain; ICP, intracranial pressure; VP, ventriculoperitoneal; LP, lumbar puncture; CSF, cerebrospinal fluid; CZA, ceftazidime-avibactam; PMB, polymyxin B; IV, intravenous.
