# Supplementary material for: Comparison of the Efficacy and Safety of Intravenous Ceftazidime-Avibactam and Intrathecal/Intraventricular Polymyxin B Sulfate in the Treatment of CNS Infections Caused by KPC-Kp in Neurosurgical Patients: A Single-Center Prospective Observational Study
Source: Antibiotics (Basel). 2026 May 13;15(5):492. doi: 10.3390/antibiotics15050492 (PMC13203131; doi:10.3390/antibiotics15050492)
Supplement: Supplementary file 1 [file antibiotics-15-00492-s001.zip › new-Supplementary Table S2.pdf]

Supplementary Table S2. Univariate Logistic Regression Analysis of Factors Associated with Clinical Cure

Variables	Odds Ratio (OR)	95% Confidence Interval (CI)	P-value
Treatment Group (CZA vs. PMB)	9.75	1.54-61.87	0.016
Admission mRS (≥4 vs. ≤3)	0.14	0.02-0.81	0.028
CSF Lactate (per 1 mmol/L increase)	0.88	0.74-1.05	0.157

Abbreviations: CZA, ceftazidime-avibactam; PMB, polymyxin B; mRS, modified Rankin Scale; CSF, cerebrospinal fluid; CI, confidence interval; OR, odds ratio.Univariate analyses were performed using logistic regression with clinical cure as the dependent variable. The results indicate that treatment group and admission mRS were significant predictors in univariate models, while CSF lactate was not.
