# Supplementary material for: Comparison of the Efficacy and Safety of Intravenous Ceftazidime-Avibactam and Intrathecal/Intraventricular Polymyxin B Sulfate in the Treatment of CNS Infections Caused by KPC-Kp in Neurosurgical Patients: A Single-Center Prospective Observational Study
Source: Antibiotics (Basel). 2026 May 13;15(5):492. doi: 10.3390/antibiotics15050492 (PMC13203131; doi:10.3390/antibiotics15050492)
Supplement: Supplementary file 1 [file antibiotics-15-00492-s001.zip › new-Supplementary Table S3.pdf]

Supplementary Table S3. CSF concentrations of polymyxin B following different administration routes

Time point (h)	Pre-dose	After-2h	After-4h	After-6h	After-8h	After-10h	After-12h
Patient A with Intraventricular injection	3.3	56.3	28.8	13.7	10.5	8.3	4.8
Patient B with Intrathecal Injection	6.6	88.1	39.3	37.4	33.1	19.9	16.2
Control	0.26	0.31	0.41	0.29	0.5	0.46	0.33

Concentrations are presented as measured values without dose normalisation.

Abbreviations: pre-dose, before drug administration; h, hours.
