# Supplementary material for: Comparison of the Efficacy and Safety of Intravenous Ceftazidime-Avibactam and Intrathecal/Intraventricular Polymyxin B Sulfate in the Treatment of CNS Infections Caused by KPC-Kp in Neurosurgical Patients: A Single-Center Prospective Observational Study
Source: Antibiotics (Basel). 2026 May 13;15(5):492. doi: 10.3390/antibiotics15050492 (PMC13203131; doi:10.3390/antibiotics15050492)
Supplement: Supplementary file 1 [file antibiotics-15-00492-s001.zip › new-Supplementary Table S4.pdf]

Supplementary Table S4. Polymyxin B Sulfate (PBS) Dosing Regimens in Included Patients

Patient ID	Sex/Age	PBS Regimen
KP1	M/51	5 mg daily × 15 days
KP5	M/54	5 mg daily × 11 days
KP8	M/25	5 mg daily × 6 days
KP9	M/30	5 mg daily × 9 days
KP10	M/64	5 mg daily × 6 days
KP11	M/18	5 mg daily × 12 days
KP12	M/57	5 mg daily × 3 days
KP15	M/39	5 mg daily × 5 days
KP16	M/49	5 mg daily × 5 days
KP17	M/30	5 mg daily × 3 days

Abbreviations: PBS,Polymyxin B Sulfate; M,Male; F,Female; mg,milligram; d, day(s).
